# Policy-Guided Model Predictive Path Integral for Safe Manipulator Trajectory Planning

**DOI:** 10.3390/s26072074

**Published:** 2026-03-26

**Authors:** Liang Liang, Chengdong Wu, Xiaofeng Wang

**Affiliations:** 1 College of Information Science and Engineering, Northeastern University, Shenyang 110819, China; 2 SIASUN Robot & Automation Co., Ltd., Shenyang 110168, China; wangxiaofeng@siasun.com; 3 Faculty of Robot Science and Engineering, Northeastern University, Shenyang 110169, China; wuchengdong@mail.neu.edu.cn

**Keywords:** manipulator trajectory planning, model predictive path integral, reinforcement learning, control barrier function, configuration-space distance field

## Abstract

**Highlights:**

**What are the main findings?**

- The proposed PG-MPPI framework integrates CD-SAC offline learning, MPPI online planning and CBF safety filtering to construct a three-level safety system, which addresses the challenges of difficult hard-constraint enforcement and weak environmental generalization in manipulator trajectory planning.
- The algorithm achieves a 100% success rate of collision-free target reaching in multi-scenario simulations on the SIASUN T12B manipulator, with the trajectory satisfying physical constraints and demonstrating excellent adaptability.

**What are the implications of the main findings?**

- The algorithm provides a new paradigm integrating global prior learning and local real-time optimization for manipulator trajectory planning, breaking through the limitations of traditional methods in sampling efficiency and constraint handling.
- Its multi-level safety assurance mechanism can be transferred to practical industrial scenarios, providing technical support for the application of industrial robots in complex scenarios such as flexible manufacturing and human–robot collaboration.

**Abstract:**

Aiming at the problems of difficult hard-constraint enforcement and weak environmental generalization ability in the safe trajectory planning of manipulators in complex environments, a Policy-Guided Model Predictive Path Integral (PG-MPPI) planning framework is proposed. This framework integrates the advantages of reinforcement learning and model predictive control to construct a global prior guidance, local real-time optimization and hard-constraint safety assurance: a Constraint-Discounted Soft Actor–Critic (CD-SAC) offline learning policy is designed, which incorporates the configuration-space distance field as a safety guidance term to realize the learning of obstacle avoidance behavior; the offline policy is used to guide the online sampling and optimization of MPPI, improving sampling efficiency and planning quality; and a Control Barrier Function (CBF) safety filter is introduced to revise control commands in real time, ensuring the strict satisfaction of constraints. Taking the SIASUN T12B manipulator as the research object, simulation comparison experiments are carried out in multi-obstacle scenarios. The results show that the PG-MPPI algorithm outperforms the comparison algorithms in the success rate of collision-free target reaching, ensures the smoothness and feasibility of the trajectory, and has a good adaptive capacity to complex environments with unknown obstacle configurations, thus providing an efficient solution for the autonomous and safe operation of manipulators.

## 1. Introduction

With the development of industrial automation towards flexible manufacturing and human–robot collaboration, manipulators are required to perform tasks in highly unstructured environments [1]. This demands that motion planning algorithms not only have real-time obstacle avoidance capability but also ensure trajectory smoothness and time optimality while satisfying complex constraints [2]. Traditional sampling-based methods, such as RRT* and PRM, can guarantee probabilistic completeness yet suffer from low search efficiency in high-dimensional spaces and fail to satisfy the millisecond-level requirement for online re-planning [3]. Optimization-based methods, like CHOMP and TrajOpt, yield high-quality trajectories but are prone to falling into local minima and have limited ability to handle non-convex obstacles [4].

Model Predictive Control (MPC) has emerged as a powerful tool to address this problem due to its capability in handling multivariable constraints and receding horizon optimization [5]. In particular, the sampling-based Model Predictive Path Integral (MPPI) leverages the large-scale parallel computing capability of Graphics Processing Units (GPUs) to effectively handle non-smooth and non-convex cost functions by applying random perturbations to control sequences and performing weighted averaging [6]. Compared with gradient-based MPC, MPPI is not restricted by the differentiability of cost functions and exhibits stronger robustness [7]. However, the performance of standard MPPI is highly dependent on the quality of the nominal control sequence and coverage of the sampling distribution. When confronted with long-horizon planning or complex geometric traps in the environment (e.g., U-shaped obstacles or narrow passages), simple mean initialization often leads to extremely low sampling efficiency, and the algorithm is likely to converge to suboptimal solutions or even fail to find feasible ones [8]. In addition, how to strictly enforce hard safety constraints while preserving sampling diversity remains a major challenge for MPPI [9].

Deep Reinforcement Learning (RL) can learn policies and value functions with a global perspective through offline training and enables extremely fast inference [10]. Nevertheless, pure end-to-end RL policies often lack adaptability to environmental changes and fail to provide rigorous hard safety guarantees [11]. To combine the accuracy of planning algorithms with the speed of learning algorithms, recent studies have begun to explore hybrid architectures of RL and MPC. A mainstream approach is to use the learned policy as a warm start for MPC to guide the search process [12,13]. For example, TD-MPC [14] integrates model learning and planning in the latent space; RL-Driven MPPI [8] directly takes the trajectory generated by the offline policy as the mean of MPPI, which significantly accelerates the convergence speed of online planning; Residual-MPPI [15] integrates residual reinforcement learning into the MPPI framework to adapt maximum entropy priors, enabling online policy customization for continuous control tasks and rapid adaptation to environmental changes without complex parameter tuning; and CoRL-MPPI [16] further utilizes a pre-trained RL policy to optimize the sampling distribution of MPPI, enhancing safety and improving the efficiency of distributed multi-robot collision avoidance in complex environments.

To provide precise collision costs for MPPI, an accurate geometric representation of the environment is crucial. However, when addressing configuration-space safety for high-dimensional manipulators, existing hybrid methods often still rely on simplified Signed Distance Functions (SDF) [17] or sparse collision penalties, overlooking the topological complexity of the joint space. To address this issue, Configuration-space Distance Fields (CDFs) have been proposed [18,19]. Unlike traditional SDFs, CDF defines the metric directly in the joint space and is, therefore, able to capture the intrinsic proximity structure of the manipulator’s configuration space. Incorporating CDF into RL reward shaping or the cost function of MPC provides smoother and more globally informative safety gradients [19], effectively guiding the sampling process away from local minima in configuration space and thereby mitigating the geometric limitations of conventional Euclidean distance fields.

In terms of safety constraint handling, Control Barrier Functions (CBFs) provide a theoretically rigorous safety assurance mechanism [20,21]. By defining the safe set as an invariant set of the state space, CBF can act as a filter to revise the nominal control law. However, the construction of CBF often relies on accurate models and gradients, and its design in high-dimensional non-convex obstacle environments is extremely challenging. When handling safety constraints for high-dimensional manipulators, a core challenge is how to ensure safety while preventing the optimization process from getting trapped in local minima. Traditional hard-constraint methods are prone to infeasible solutions or even oscillatory behavior in complex non-convex environments. Recent studies have, therefore, shifted toward more flexible soft-constraint mechanisms. Constraint-Discounted MPPI (CD-MPPI) [22] introduces a constraint-violation-aware discount factor, which achieves “soft termination” by dynamically adjusting the weighting of future returns and has demonstrated superior stability in safe learning. CaT [23] reformulates constraints as stochastic terminations and dynamically truncates trajectory returns according to the degree of constraint violation, thereby not only simplifying reward engineering but also enabling efficient sim-to-real transfer through curriculum learning. These methods provide MPPI with smoother gradient guidance and help avoid the myopic behavior often induced by hard constraints.

Based on the above background, this paper proposes a Policy-Guided Model Predictive Path Integral (PG-MPPI) control framework aiming to solve the problem of safe motion planning for high-dimensional manipulators in complex environments. The main contributions of this paper are as follows:

1. A dense safety-guided policy learning method based on CDF is proposed. To overcome the limitations of sparse collision penalties of traditional workspace signed distance functions, CDF is integrated into the design of the SAC reward function, and its continuously differentiable gradient characteristic is utilized to provide global dense guidance for obstacle avoidance behavior;
2. A policy-guided MPPI online planning architecture is designed to achieve deep integration of global prior and local optimization. The offline-trained Constraint-Discounted SAC (CD-SAC) policy is used as the nominal control sequence generator for MPPI to provide a high-quality warm start, concentrating the sampling distribution near the global optimal solution. This design alleviates the shortcomings of traditional MPPI and compensates for the generalization limitations of the offline policy via MPPI’s online receding horizon optimization;
3. A multi-level safety system featuring “policy soft guidance + optimization hard constraint + filter final guarantee” is constructed. At the offline learning layer, the safety preference is internalized into the policy through CDF and TD-CD; at the online planning layer, the trajectory feasibility is enhanced through the explicit cost penalty of MPPI; at the execution layer, a safety filter based on first-order CBF is introduced to perform real-time projection correction on control commands.

The rest of this paper is organized as follows: Section 2 presents the problem formulation and relevant preliminaries. Section 3 elaborates on the implementation details of the proposed PG-MPPI algorithm, including the design of the offline constraint-discounted reinforcement learning policy and the online planning system. Section 4 verifies the effectiveness and superiority of the algorithm through multi-scenario simulation experiments. Section 5 summarizes the overall work of this paper and prospects future research directions.

## 2. Preliminaries

### 2.1. Problem Formulation

For the trajectory planning problem of an *n*-degree-of-freedom manipulator, we define $q\in {\mathbb{R}}^{n}$ as the joint angle vector in the robot’s configuration space. The joint angles and joint velocities form the system state vector $x=[q,\dot{q}]$, and the joint acceleration $\ddot{q}$ is taken as the control input vector *u*. Joint position, velocity, and acceleration can be transformed into one another through differentiation and integration. The manipulator’s dynamic system is modeled in discrete-time form:

(1) $$x_{t+1}=f\left(x_{t},u_{t}\right),$$

where $f\left(\cdot \right)$ denotes the discrete dynamic function and *t* represents the time step.

The core objective of this study is to seek a sequence of control inputs over an infinite time horizon to minimize the cumulative cost:

(2) $$\underset{\left\{u_{0},u_{1},\ldots \right\}}{\min}\sum_{t=0}^{\infty }l\left(x_{t},u_{t}\right),$$

where $l\left(\cdot \right)$ denotes the stage cost function.

To ensure the safety, physical feasibility and motion smoothness of the manipulator’s trajectory, three types of strict core constraints are further introduced on the basis of the aforementioned optimal control objective, forming a constrained optimal control problem:

1. Obstacle avoidance constraint: Define the minimum safety distance $d_{\mathrm{safe}}$ between the manipulator’s links, joints and obstacles in the workspace and require the actual minimum distance to satisfy the safety threshold $d\left(x_{t}\right)\geq d_{\mathrm{safe}}$ during motion to ensure no geometric collision;
2. State constraint: Set upper and lower bounds for joint angles and joint velocities, i.e., $q_{\min}\leq q\leq q_{\max}$, ${\dot{q}}_{\min}\leq \dot{q}\leq {\dot{q}}_{\max}$, to prevent the system from entering a physically infeasible state for the mechanical structure and drive system;
3. Control constraint: Set an amplitude upper bound for joint acceleration, i.e., $\left\|u\right\|\leq u_{\max}$, to match the actual output capability of actuators and prevent mechanical vibration, impact or hardware damage caused by abrupt changes in control signals.

The above constrained infinite-horizon optimal control model constitutes the basic mathematical framework for safe trajectory planning of manipulators.

### 2.2. Model Predictive Path Integral

MPPI is a class of sampling-based model predictive control methods [6]. Its core idea is to perform random sampling and search around the nominal control sequence, abandoning the traditional approach of selecting a single optimal trajectory. This method is suitable for trajectory planning scenarios of nonlinear and high-dimensional systems.

For the initial nominal control sequence $\bar{U}=\left\{{\bar{u}}_{0},\ldots ,{\bar{u}}_{H}\right\}$ within the receding horizon *H*, an independent zero-mean Gaussian noise sequence is generated for each of the *K* sampled trajectories at time *t*:

(3) $$\varepsilon_{i}^{k}∼N\left(0,\Sigma \right),$$

where i=0,…,H−1, k=1,…,K, and Σ denote the noise covariance matrix. Based on this, the candidate control sequence for the *k*-th trajectory is constructed as

(4) $$u_{i}^{k}={\bar{u}}_{i}+\varepsilon_{i}^{k}.$$

A forward rollout is executed for each candidate control sequence, and the corresponding state trajectory ${\left\{x_{t+i}^{k}\right\}}_{t=0}^{H}$ is obtained by combining with (1). The cumulative cost over the receding horizon is given by

(5) $$S_{k}=S\left(U^{k};x_{t}^{k}\right)=\sum_{i=0}^{H-1}l\left(x_{t+i}^{k},u_{t+i}^{k}\right)+l_{f}\left(x_{t+H}^{k}\right),$$

where $U=\left\{u_{t},\ldots ,u_{t+H-1}\right\}$ is the control sequence, $l\left(\cdot \right)$ is the stage cost function, and $l_{f}\left(\cdot \right)$ is the terminal cost function. An exponential transformation is applied to the cumulative cost of each sampled trajectory to construct an exponential weight:

(6) $$\omega_{k}=\exp\left(-\frac{1}{\lambda }\left(S_{k}-S_{\min}\right)\right),$$

where λ>0 is the temperature parameter. A smaller value of λ results in a stronger bias of the weight toward low-cost trajectories, whereas a larger value enhances the exploratory nature of sampling. The normalized weights ${\tilde{\omega }}_{k}$ are used to perform a weighted average of all sampled noise sequences, thus realizing the update of the initial nominal control sequence:

(7) $${\bar{u}}_{i}\leftarrow u_{i}+\sum_{k=1}^{K}{\tilde{\omega }}_{k}\varepsilon_{i}^{k}.$$

MPPI adopts a receding horizon execution strategy, where only the control signal $u_{t}^{*}={\bar{u}}_{0}$ at the initial time step of the updated nominal control sequence is extracted and applied to the manipulator’s actuators to drive the robot to complete the motion of the current time step. When proceeding to the next time step *t* + 1, a shifting operation needs to be performed on the control sequence.

### 2.3. Soft Actor–Critic

The core objective of reinforcement learning is to learn an approximately optimal policy π that enables an agent to maximize the long-term expected return during its interactions with the environment. Soft Actor–Critic (SAC) is an off-policy actor–critic algorithm based on the maximum entropy reinforcement learning framework [24], which maximizes both the expected cumulative return and policy entropy simultaneously, formulated as

(8) $$\underset{\pi }{\max}E_{\tau \sim \pi }\left[\sum_{t=0}^{\infty }\gamma^{t}\left(r\left(s_{t},a_{t}\right)+\alpha H\left(\pi \left(\cdot |s_{t}\right)\right)\right)\right],$$

where $\gamma \in \left[0,1\right)$ is the discount factor and $r\left(s_{t},a_{t}\right)$ is the immediate reward obtained by the agent executing action $a_{t}$ in state $s_{t}$. A larger entropy value $H\left(\pi \left(\cdot |s_{t}\right)\right)=-E_{a∼\pi \left(\cdot |s_{t}\right)}\left[\log\pi \left(a|s_{t}\right)\right]$ means the policy has stronger exploratory properties. α>0 is the temperature parameter.

SAC adopts a twin Q-networks to approximate the state-action value function, so as to alleviate the value overestimation problem of the Q-networks. For samples $\left(s_{t},a_{t},r_{t},s_{t+1},d_{t}\right)$, where $d_{t}$ is the termination flag in the replay buffer D, the soft Bellman backup target is defined as

(9) $$y_{t}=r_{t}+\gamma \left(1-d_{t}\right)E_{a_{t+1}∼\pi_{\phi }\left(\cdot |s_{t+1}\right)}\left[\underset{i\in \left\{1,2\right\}}{\min}Q_{{\bar{\theta }}_{i}}\left(s_{t+1},a_{t+1}\right)-\alpha \log\pi_{\phi }\left(a_{t+1}|s_{t+1}\right)\right],$$

where ϕ denotes the learnable parameters of the actor network.

The training objective of the critic network is to minimize the mean squared error of the temporal difference (TD) error, given by

(10) $$\underset{\theta_{i}}{\min}E_{(s_{t},a_{t},r_{t},s_{t+1},d_{t})\sim D}\left[{\left(Q_{\theta_{i}}\left(s_{t},a_{t}\right)-y_{t}\right)}^{2}\right],$$

where $\theta_{i}$ denotes the parameters of the target critic network.

The actor network adopts a stochastic policy formulation and outputs the probability distribution of all actions under a given state s. Its core training objective is to maximize the expected soft value function. The objective function for policy update is given by

(11) $$\underset{\phi }{\min}E_{s_{t}\sim D,a_{t}\sim \pi_{\phi }}\left[\alpha \log\pi_{\phi }\left(a_{t}|s_{t}\right)-\underset{i\in \left\{1,2\right\}}{\min}Q_{\theta_{i}}\left(s_{t},a_{t}\right)\right].$$

In SAC, the temperature parameter α is treated as a learnable parameter and updated by maximizing the entropy-weighted expected return objective. Based on the aforementioned principles, SAC leverages neural networks to learn the optimal policy, which satisfies the maximization condition of the objective function (8).

## 3. Policy-Guided MPPI

As discussed in Section 2, RL learns a parameterized policy by solving Equation (8) offline using samples collected from interactions with the environment. The learned RL policy exhibits excellent real-time performance in the online decision-making process yet may fail in certain states due to suboptimality during training and rare state visits. In contrast, MPPI minimizes Equation (5) via rollouts starting from the current state to find the optimal control sequence at each time step. It outperforms the RL policy in ensuring policy feasibility across all feasible states but results in poor online decision-making efficiency. In this section, we propose a method that combines RL with MPPI, thereby providing better control performance for complex systems with flexible cost criteria.

### 3.1. Algorithm Framework

**Figure 1:**
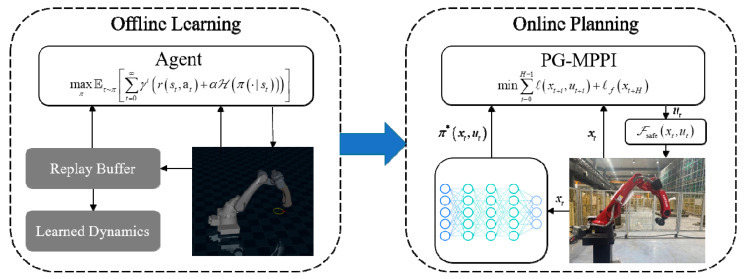
Architecture of PG-MPPI.

The proposed PG-MPPI algorithm framework integrates the global policy guidance capability of reinforcement learning with the local online fine optimization capability of MPPI to realize the globally and locally coordinated safe trajectory planning of manipulators in complex environments. On the whole, it forms a closed-loop control system featuring global prior guidance, local real-time optimization and hard safety constraint guarantee. The algorithm architecture is shown in Figure 1.

This framework is mainly divided into two modules, which are an offline learning phase and an online planning phase:

1. Offline Learning: A prior controller is obtained through repeated interactions of reinforcement learning in a simulation environment. The objectives of target reaching + safety constraints are encoded into the reward function or constraint discounting mechanism to train a policy $\pi_{\phi }\left(a|s\right)$. Such a policy is capable of providing action guidance with a long planning horizon and shifting a large amount of computational load from the online execution phase to the offline training phase;
2. Online Planning: Real-time optimization is performed under the current actual state and environment to ensure safe execution of the manipulator. The output of the offline policy is adopted as the nominal control sequence of MPPI; random sampling and rollout are conducted around this sequence. The desired control signal is then revised by a safety filter to satisfy all safety constraints. This approach not only leverages the RL prior to improve sampling efficiency but also utilizes the explicit cost function and receding horizon optimization of MPPI.

### 3.2. Offline Policy Learning

With the precise positioning of the manipulator’s end-effector at the designated target as the core primary task, the Configuration-space Distance Field (CDF) is adopted as the core safety guidance term. Its gradient information and a safety penalty mechanism are integrated into the design of the reinforcement learning reward function, guiding the policy to actively learn obstacle avoidance behaviors during offline training. This enables the manipulator to autonomously keep away from obstacle collision areas, achieving the collaborative optimization of task completion and safe obstacle avoidance.

#### 3.2.1. Reward Design

Let the Euclidean distance between the manipulator’s end-effector $x\left(q_{t}\right)$ and the target position $x_{g}$ be expressed as

(12) $$d_{t}={\left\|x\left(q_{t}\right)-x_{g}\right\|}_{2}.$$

Based on this distance, a progress-based target reward is designed to make the reward signal change continuously as the end-effector approaches the target, providing a stable gradient guidance for policy optimization, with the form

(13) $$r_{\mathrm{goal}}\left(t\right)=k_{g}\left(d_{t-1}-d_{t}\right),$$

where $k_{g}$ denotes the reward weight, which is used to adjust the proportion of the primary task in the reward function.

Traditional obstacle modeling usually employs the SDF $f_{s}$ to represent the distance between a point p in space and the robot’s surface $\partial r\left(q\right)$:

(14) $$f_{s}\left(p,q\right)=\pm \underset{p^{\prime }\in \partial r\left(q\right)}{\min}\left\|p-p^{\prime }\right\|,$$

where ± is a sign indicating the distance direction, which is positive if the point is outside and negative if inside. Such models only treat collisions as hard constraints or simple soft penalties, increasing the cost when a collision is detected. This approach fails to fully utilize the obstacle distance information to provide active guidance for collision-free path planning, thus leading to low learning efficiency of obstacle avoidance policies.

To address the above problems, this study adopts the Configuration-space Distance Field (CDF) for obstacle modeling [19]. Taking joint values as independent variables, this function represents the minimum distance from the manipulator’s current joint configuration to the critical joint configuration where a collision occurs, and this distance determines the contact between the robot and the obstacle:

(15) $$f_{c}\left(p,q\right)=\underset{q^{\prime }}{\min}\left\|q-q^{\prime }\right\|,$$

where $q^{\prime }$ satisfies collision constraints, and the configuration set corresponding to a given point p can be found on the zero-level set of the SDF model via inverse kinematics. For multi-obstacle scenarios, the global safety CDF is defined as the minimum single-obstacle CDF value with respect to the zero level sets of all obstacles, corresponding to the most critical obstacle. In an *n*-dimensional configuration space with *M* obstacles, the computational complexity is $O=\left(M\cdot n\right)$, without suffering from the curse of dimensionality.

The CDF is continuous and differentiable almost everywhere in the configuration space, and its gradient naturally points to the locally optimal collision escape direction. Neural CDF [19] approximates the CDF function using a multi-layer perceptron, and its gradient $\nabla c\left(q\right)$ can be computed directly through automatic differentiation in the neural network, with computation time on the order of microseconds.

**Figure 2:**
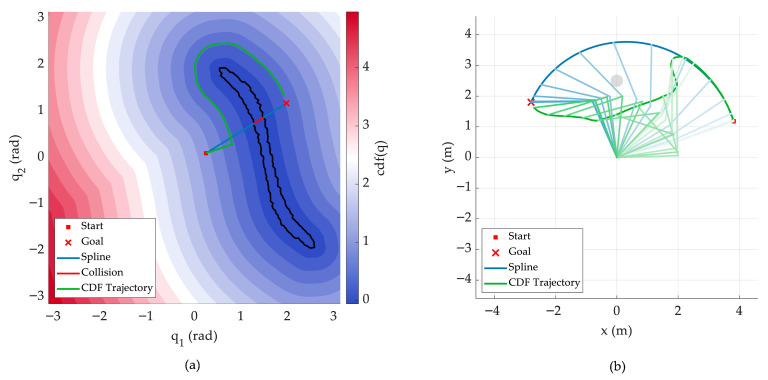
Schematic of CDF-based 2-DOF trajectory. (**a**) CDF contour lines and trajectory in the configuration space. The black contour line is the set of configurations in contact with obstacles. Motion along the tangent and gradient of the contour lines can bypass obstacles. (**b**) Workspace trajectory corresponding to the configuration space trajectory.

It can provide a direct gradient guidance signal for obstacle avoidance behavior learning, making it an ideal safety guidance term for manipulator joint space trajectory planning. The principle is shown in Figure 2.

A nonlinear safety penalty term is designed based on the CDF to realize adaptive punishment for collision risks, with the form

(16) $$r_{\mathrm{safe}}\left(t\right)=-\mu {\left[\max\left(0,d_{m}-f_{c}\left(q_{t}\right)\right)\right]}^{p},$$

where μ>0 is the safety penalty weight, p≥1 is the penalty exponent, and $d_{m}>0$ is the safety margin threshold. When the manipulator’s joint configuration is in the safe region $f_{c}\left(q_{t}\right)\geq d_{m}$, the safety penalty term is 0 and does not interfere with the execution of the primary task. When the joint configuration enters the low-CDF risk region $f_{c}\left(q_{t}\right)<d_{m}$, the penalty value increases nonlinearly with the decrease in the CDF value. A larger penalty exponent results in a higher penalty intensity in the collision risk region, which can quickly push the policy back to the safe region while avoiding excessive punishment for minor boundary crossings.

To further strengthen safety constraints, the current training episode is terminated immediately, and a one-time collision penalty is imposed when an actual collision occurs to the manipulator. Combining the target reward, safety penalty term and one-time collision penalty, the total reward function is obtained as

(17) $$r_{t}=r_{\mathrm{goal}}\left(t\right)+r_{\mathrm{safe}}\left(t\right)+r_{\mathrm{col}}\left(t\right).$$

This reward function achieves an organic integration of the primary task objective and safety obstacle avoidance constraints. It not only drives the manipulator to complete the end-effector positioning task through the progress-based reward but also enables the policy to actively learn obstacle avoidance behaviors during offline training by virtue of the CDF’s gradient guidance and nonlinear penalty mechanism, thus ensuring the task feasibility and collision-free safety of the trajectory from the perspective of reward design.

#### 3.2.2. Constraints Based on Discount Factor

To achieve unified and adaptive management of multiple types of constraints in manipulator trajectory planning, this section converts the degree of constraint violation into a reward discount weight through a random termination signal and combines the Exponential Moving Average (EMA) and finite-horizon discount rules to form a constraint handling scheme that balances safety and control continuity.

Various physical and safety constraints during the manipulator’s motion are uniformly abstracted into a general form of inequality constraints, covering all hard constraints such as joint position boundaries and the amplitude limits of joint velocity and acceleration:

(18) $$\begin{array}{cc} c_{i}\left(x,u\right)\leq 0 & \forall i\in I \end{array}.$$

To avoid the threshold dependence and risks of constraint violation superposition associated with manually designed penalty functions, this section adopts a random termination signal based on constraint violation to directly map the degree of violation to a discount weight for future rewards, which is defined as

(19) $$\delta_{t}=\underset{i\in I}{\max}\left(p_{i}^{\max}\cdot \mathrm{clip}\left(\frac{{\left[c_{t}\right]}_{i}}{c_{i}^{\max}},0,1\right)\right),$$

where $p_{i}^{\max}\in \left[0,1\right]$ is the maximum termination probability parameter for *i*-th constraint, ${\left[c_{t}\right]}_{i}$ is the actual violation amount of constraint *i* at step *t*, and $c_{i}^{\max}$ is the maximum violation value of the *i*-th constraint across all trajectory samples. Equation (19) has the following properties:

1. Constraint violation no longer relies on manually designed penalty functions. Instead, the degree of violation is directly converted into a discount weight for future rewards. The more severe the violation, the closer the value $\delta_{t}$ is to $p_{i}^{\max}$, and the higher the proportion of future rewards being discounted;
2. A high termination probability is triggered as long as one constraint is severely violated, which prevents safety risks or motion failure caused by the superposition of multiple minor violations and ensures the stringency of constraints;
3. In the absence of violations, ${\left[c_{t}\right]}_{i}=0$ and $\delta_{t}=0$, meaning future rewards are not discounted to encourage normal exploration. In the case of minor violations, $0<{\left[c_{t}\right]}_{i}<c_{t}^{\max}$ and $\delta_{t}\in \left(0,p_{i}^{\max}\right)$, future rewards are partially discounted, which not only warns of violations but also allows the robot to learn recovery strategies. In the case of severe violations, ${\left[c_{t}\right]}_{i}\geq c_{t}^{\max}$ and $\delta_{t}=p_{i}^{\max}$, which directly terminates all future rewards and strictly prohibits severe violations.

To avoid fluctuations in the discount factor caused by extreme violation values in a single training batch and to realize data-driven adaptive learning of the violation reference range, an EMA mechanism is adopted to update the historical maximum violation value of each constraint $c_{i}^{\max}$:

(20) $$c_{i}^{\max}\leftarrow \tau_{c}\cdot {\hat{c}}_{i}^{\max}+\left(1-\tau_{c}\right)\cdot c_{i}^{\max},$$

where ${\hat{c}}_{i}^{\max}=\max_{t}{\left[c_{t}\right]}_{i}$ is the maximum violation value of the *i*-th constraint at all time steps in the current training batch and $\tau_{c}\in \left[0,1\right]$ is the decay rate parameter representing the percentage of historical information retained. This mechanism enables the violation reference value to be dynamically adjusted with the training process, quickly adapting to the actual range of environmental constraints in the early stage and tending to be stable in the later stage.

#### 3.2.3. Constraint-Discounted SAC Learning Strategy

Based on the aforementioned constraint discounting mechanism and combined with the maximum entropy reinforcement learning framework of the SAC algorithm, CD-SAC learning strategy is adopted. Constraint information is injected into the temporal-difference backup process of SAC in the form of soft termination, enabling multi-objective fusion learning for primary task reward, safety guidance, and constraint discounting.

The agent performs rollout sampling for state transitions $\left(s_{t},u_{t},r_{t},s_{t+1}\right)$ in a constrained environment and maps the degree of constraint violation at each step to a soft termination intensity $\delta_{t}$ according to Equation (19), thereby deriving a time-varying discount factor $\gamma_{t}$:

(21) $$\gamma_{t}=\gamma \left(1-\mathrm{clip}\left(\delta_{t},0,1\right)\right),$$

where γ denotes the baseline discount factor of SAC. When a constraint violation occurs or is imminent, $\gamma_{t}$ is reduced, thus weakening the propagation of future rewards across unsafe state transitions and achieving soft termination.

In the critic network update, the twin Q-networks structure and entropy regularization form of SAC remain unchanged, with only the constant discount factor in the Temporal-Difference (TD) target replaced by the time-varying $\gamma_{t}$. For mini-batches $\left\{\left(s_{t},u_{t},r_{t},s_{t+1},\gamma_{t}\right)\right\}$ sampled from the replay buffer, a soft value function target is constructed as follows:

(22) $$V\left(s_{t+1}\right)=E_{a_{t+1}∼\pi \left(\cdot |s_{t+1}\right)}\left[\min\left(Q_{{\bar{\theta }}_{1}}\left(s_{t+1},a\right),Q_{{\bar{\theta }}_{2}}\left(s_{t+1},a\right)\right)-\alpha \log\pi \left(a|s_{t+1}\right)\right].$$

The Temporal-Difference Constraint-Discounting (TD-CD) target is then given by

(23) $$y_{t}=r_{t}+\gamma_{t}V\left(s_{t+1}\right).$$

The update rules for the critic network, actor network and temperature parameter all follow the standard SAC formulation, and the overall training process remains an online interactive off-policy learning paradigm. The only difference is that the replay buffer additionally stores the $\gamma_{t}$ value for each state transition, thus injecting constraint information into the TD backup of the critic network.

In contrast to hard reward penalties for constraint violations, TD-CD exerts the effect of constraints on the value propagation path: the discount factor is automatically reduced when constraint violations occur or their severity increases, which attenuates the impact of subsequent future rewards on the current state-action value and mitigates the overestimation of value and gradient amplification by the policy in unsafe regions. This mechanism does not rely on on-policy trajectories and is naturally compatible with experience replay buffers, resulting in smoother and more stable training.

### 3.3. Online Trajectory Planning

The offline-trained CD-SAC policy can provide global and long-horizon action guidance for manipulator planning and avoid static obstacles in the training scenarios, but it lacks the local adaptability to unmodeled obstacles. To address this issue, this paper fuses this offline policy with the local online optimization capability of MPPI and introduces a CBF safety filter.

#### 3.3.1. CBF-Based Safety Filter

The safety filter serves as the final safety barrier in the online planning phase, whose core function is to revise the desired control commands output by MPPI in real time. This ensures that the final control signals applied to the manipulator strictly satisfy the hard constraints of obstacle avoidance, state and control, thus guaranteeing the system’s safety from a theoretical perspective.

Taking the first-order Control Barrier Function (CBF) as the core [20], this study designs a safety filter at the velocity level to achieve real-time collision risk avoidance and feasible control signal projection. When defining the robot configuration, let $p_{i}\left(q\right)$ be the end point of link *i*. To quantify the safety margin between the manipulator and obstacles, the minimum margin function is defined as

(24) $$c\left(q\right)=\underset{j}{\min}\underset{i\in \left\{0,\ldots ,n-1\right\}}{\min}\left(\mathrm{dist}\left(o_{j},\left[p_{i}\left(q\right),p_{i+1}\left(q\right)\right]\right)-{\tilde{r}}_{j}\right),$$

where $\mathrm{dist}\left(o,\left[a,b\right]\right)$ denotes the Euclidean distance from a point o to a line segment $\left[a,b\right]$ and ${\tilde{r}}_{j}$ is the preset minimum safety distance. Therefore, a geometric collision occurs between the manipulator and obstacles when $c\left(q\right)<0$; the manipulator is in the safe region when $c\left(q\right)\geq 0$; and a larger value of $c\left(q\right)$ indicates a higher safety margin.

Treating $c\left(q\right)$ as the safety function, we require that the time derivative of the safety function satisfies a non-negativity constraint. This ensures that the manipulator always moves in the direction of increasing safety margin when starting from a safe state, avoiding entry into collision areas:

(25) $$\dot{c}\left(q\right)=\nabla c{\left(q\right)}^{T}\dot{q}\geq -\alpha \left(c\left(q\right)\right),$$

where $\alpha \left(\cdot \right)$ is a class K function (a linear function $\alpha \left(c\right)=\rho c$, ρ>0 is adopted in this study).

Its role is to constrain the decay rate of the safety function: when $c\left(q\right)$ approaches 0, $\dot{c}\left(q\right)\geq 0$ is forced to be positive, making the manipulator move away from collision areas rapidly.

The essence of the safety filter is a Quadratic Programming (QP) problem with linear inequality constraints [25]. The objective is to find a feasible joint velocity ${\dot{q}}_{\mathrm{safe}}$ that is closest to the desired joint velocity ${\dot{q}}_{\mathrm{des}}$ by MPPI, under the premise of satisfying the CBF constraints and the physical constraints of joint velocity. The constructed QP optimization problem is as follows:

(26) $${\dot{q}}_{\mathrm{safe}}=\arg\underset{\dot{q}}{\min}\left\|\dot{q}-{\dot{q}}_{\mathrm{des}}\right\|,$$

(27) $$\begin{array}{cc} s.t. & \nabla c{\left(q\right)}^{T}\dot{q}+\alpha \left(c\left(q\right)\right) \end{array}\geq 0.$$

This QP problem has a closed-form solution and can be solved in real time using the Lagrangian method, thus satisfying the real-time requirement for online trajectory planning. When $\nabla c^{T}{\dot{q}}_{\mathrm{des}}\geq -\alpha \left(c\right)$, the constraint is not activated, and the solution is ${\dot{q}}_{\mathrm{safe}}={\dot{q}}_{\mathrm{des}}$; when the constraint is activated, ${\dot{q}}_{\mathrm{des}}$ is orthogonally projected onto the boundary of the constraint half-space to obtain the feasible solution:

(28) $${\dot{q}}_{\mathrm{safe}}={\dot{q}}_{\mathrm{des}}-\frac{\alpha \left(c\right)+\nabla c{\left(q\right)}^{T}{\dot{q}}_{\mathrm{des}}}{{\left\|\nabla c\left(q\right)\right\|}_{2}^{2}+\delta }\nabla c\left(q\right),$$

where δ>0 is a numerical stabilization term.

#### 3.3.2. Algorithm Implementation

PG-MPPI integrates MPPI with a learned policy to enable safe and efficient planning. The overall implementation steps are presented in Algorithm 1.
**Algorithm 1** PG-MPPI Pseudocode**Required:** Dynamics $x_{t+1}=f\left(x_{t},u_{t}\right);\mathrm{horizon}\;H;\mathrm{samples}\;K;\mathrm{temperature}\lambda ;\mathrm{noise}\;\mathrm{covariance}\Sigma ;\mathrm{stage}\;\mathrm{cost}\;l\left(x,u\right);\mathrm{terminal}\;\mathrm{cost}\;l_{f}\left(x,u\right);$ learned policy
$\pi_{\phi }\left(a|s\right);\mathrm{control}\;\mathrm{constraint}\;\mathrm{set}\;U;\mathrm{safety}\;\mathrm{filter}\;F_{\mathrm{safe}}\left(\cdot \right).$
**Ensure:** $u_{t}$ applied to the robot at each time step.1:**Initialize** norminal control sequence $U=\left\{u_{0},\ldots ,u_{H-1}\right\}.$2:**for** t=0,1,2…,K **do**3:    Observe current state
$x_{t}.$

**Policy-guided nominal (warm start)**4:    Predict a nominal rollout under the learned policy:5:        
${\hat{x}}_{0}\leftarrow x_{t}$
6:    **for** 
i=0 to
H−1 **do**
7:        
${\hat{u}}_{i}\leftarrow E\left[\pi_{\phi }\left(\cdot |{\hat{x}}_{i}\right)\right]$
8:        
${\hat{x}}_{i+1}\leftarrow f\left({\hat{x}}_{i},{\hat{u}}_{i}\right)$
9:    **end for**
10:    Set the nominal sequence
$U\leftarrow \left\{{\hat{u}}_{0},\ldots ,{\hat{u}}_{H-1}\right\}.$

**MPPI update**11:    
$U\leftarrow \mathrm{MPPI}\left(x_{t},U;f,l,l_{f},H,K,\lambda ,\Sigma ,U\right)$

**Safety filtering and execution**12:    
$u_{t}^{*}\leftarrow u_{0}$
13:    
$u_{t}^{\mathrm{safe}}\leftarrow F_{\mathrm{safe}}\left(x_{t},u_{t}^{*}\right)$
14:    Apply
$u_{t}^{\mathrm{safe}}$ to the robot, obtain next state
$x_{t+1}.$

**Receding horizon shift**15:    
$U\leftarrow \left\{u_{1},u_{2},\ldots ,u_{H-1},u_{H-1}\right\}$
16:**end for**

In the offline phase, a policy $\pi_{\phi }\left(a|x\right)$ is trained in a simulation environment, where a CDF-based safety term guides the policy to learn obstacle avoidance and keep away from collision-prone regions. In the online phase, firstly, the CD-SAC policy is used to perform forward rollout to generate a nominal control sequence within the receding horizon *H*, which is then set as the initial nominal control sequence for MPPI. Next, following the standard MPPI update algorithm, the processes of sampling, rollout, cost calculation and weighted update are executed to obtain an optimized control sequence. The control signal at the initial time step of the optimized control sequence is extracted and fed into the safety filter $F_{\mathrm{safe}}\left(x_{t},u_{0}\right)$, yielding a feasible control signal that satisfies all hard constraints. Finally, the feasible control signal is applied to the manipulator to drive the robot to complete the motion of the current time step.

Compared with traditional MPPI or RL methods, PG-MPPI integrates the advantages of both and features the following prominent characteristics:

1. Guided sampling and variance reduction. The nominal control of standard MPPI is usually based on the translation of the previous time step or simple heuristics, which is prone to falling into local minima or failing under complex constraints. PG-MPPI leverages the RL policy to provide a high-quality warm-start sequence, concentrating the sampling distribution near the globally optimal solutions. This greatly improves the sampling effectiveness and convergence speed;
2. Global planning capability and fast local correction. The RL policy learns a long-horizon value function through offline training, enabling it to handle tasks that require global information. In contrast, MPPI can perform high-frequency, real-time local correction on policy outputs, effectively responding to unencountered obstacles or model errors in offline training;
3. Complementary safety and constraint handling. RL training based on CDF enables the policy to internalize a soft safety preference, so that the output initial trajectory, even if not perfect, is always close to the safe region. On this basis, MPPI further guarantees the feasibility and safety of the trajectory at the execution level through hard-constraint projection and collision cost penalty, forming a dual assurance mechanism of policy soft guidance + optimization hard constraint.

## 4. Experiments

To verify the effectiveness and superiority of the PG-MPPI algorithm, the T12B (SIASUN Robot & Automation Co., Ltd., Shenyang, China) six-joint manipulator is taken as the experimental object. A platform is built based on the MuJoCo [26] simulation framework, and environmental obstacles are modeled using CDF [19]. Multi-scenario comparative experiments are designed to conduct comparative analyses of PG-MPPI with SF-MPPI (the standard MPPI algorithm with a safety filter) and SF-SAC (the standard SAC algorithm with a safety filter). All experiments are conducted on a workstation equipped with an Intel i7-14650HX processor and an NVIDIA RTX 4050 graphics card, with the simulation time step set to 0.01 s.

### 4.1. Offline Policy Learning

**Figure 3:**
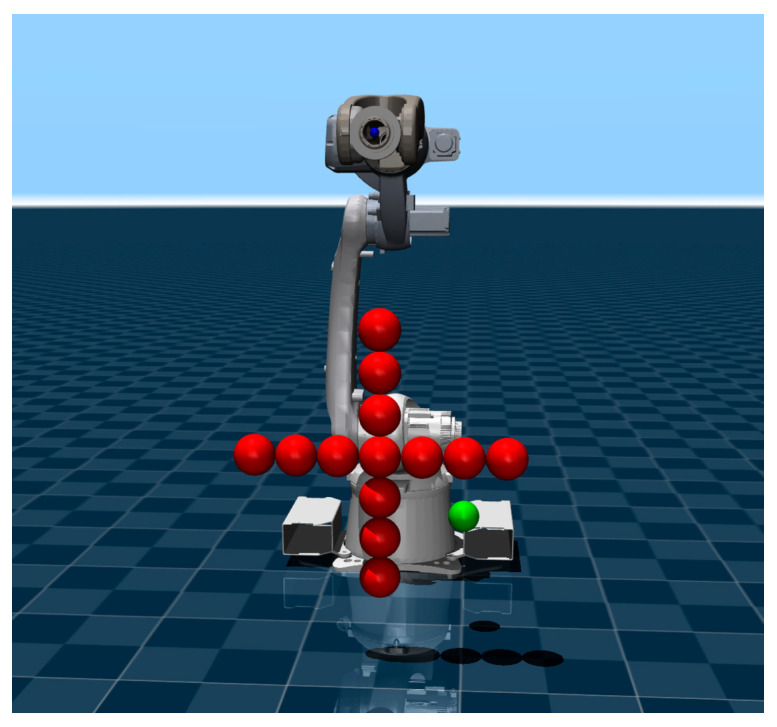
Manipulator and obstacle simulation.

The environment is shown in Figure 3, where the motion target is a green sphere with coordinates (0.6, 0.2, 0.3). A cross-shaped obstacle centered at (0.8, 0.0, 0.5) and consisting of 13 red spheres with a radius of 0.05 is present in the workspace.

Based on the simulation environment built in Section 4.1 and the designed constraint discounting mechanism, the proposed CD-SAC algorithm is adopted to conduct offline training for the actor network and critic network. The core parameters of the networks are shown in Table 1.

**Figure 4:**
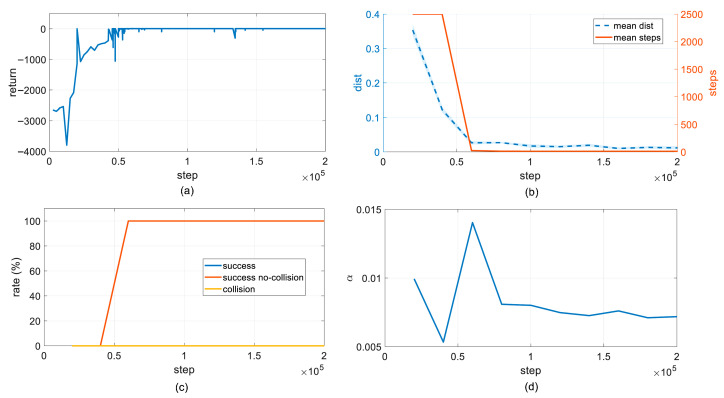
Metric curves during training. (**a**) Episode return; (**b**) mean distance; (**c**) success rate; (**d**) temperature parameter.

The evolution of key metrics during the training process is shown in Figure 4. In the initial stage of training, before the policy has effectively explored the action space, the episodic return is generally low and exhibits significant fluctuation. As iterations progress, the policy performance improves rapidly, with the episodic return climbing steadily from a significantly negative range and converging after approximately 50,000 steps. This indicates that CD-SAC has learned a control policy capable of efficiently completing the end-effector positioning task with low cost.

The evolution of quantitative metrics during training is illustrated in Figure 4. The average distance between the end-effector and the target (eval mean distance) starts at approximately 0.35 m in the initial stage and exhibits an exponential and rapid decrease as the number of iterations increases, eventually stabilizing below 0.02 m after about 50,000 steps, consistently meeting the positioning accuracy requirements. Concurrently, the average number of steps per episode (eval mean steps) rapidly drops from a high level near the maximum step limit to a lower steady state, with its trend highly synchronized with the distance metric. This clearly demonstrates that the policy behavior quickly transitions from an inefficient pattern characterized by aimless exploration, failure to reach the target, and frequent timeouts to an efficient execution mode featuring concise paths and direct target arrival. This fully verifies the advantages of the CD-SAC algorithm in terms of fast convergence speed and stable steady-state performance.

In terms of safety, the collision rate (eval collision rate) remains consistently at an extremely low level close to 0 throughout the training process. Correspondingly, both the task success rate (success) and the collision-free success rate (success no-collision) synchronize to reach 100% after 50,000 steps and remain stable thereafter. These results intuitively prove that the Configuration-space Distance Field (CDF)-based safety guidance and nonlinear penalty mechanism introduced in this paper provide continuous and smooth obstacle avoidance gradients for the policy. This enables the manipulator to actively avoid hazardous areas during learning, ensuring motion safety while completing positioning tasks, thus achieving collaborative optimization of task performance and safety constraints.

Furthermore, the evolution curve of the adaptive temperature parameter reflects the dynamic balance between exploration and exploitation: after a brief resurgence in exploration intensity during the initial convergence phase, the temperature parameter gradually decreases and stabilizes around 0.007. This indicates that the CD-SAC algorithm can autonomously reduce policy entropy in the later stages of training, shifting from exploration-dominated behavior to stable exploitation, thereby locking onto a more robust optimal control policy and further ensuring the consistency and reliability of the output trajectories.

In summary, the training curves collectively verify that the proposed CD-SAC algorithm converges rapidly within a small number of training steps while achieving a 100% task success rate and near-zero collision rate. It maintains high stability after convergence, balancing execution efficiency, positioning accuracy, motion safety, and control smoothness, providing a high-quality and highly reliable global prior policy for subsequent online PG-MPPI planning.

### 4.2. Obstacle Avoidance Trajectory

Based on the converged CD-SAC policy network trained offline, this paper configures the core parameters of the PG-MPPI online planner, with the specific values presented in Table 2. The hyperparameters were selected by balancing goal-reaching accuracy, sampling diversity, control smoothness, and online computational efficiency. In the real-time execution phase, the algorithm adopts a two-layer time-scale architecture: the optimal policy obtained offline serves as the global prior and is updated in a rolling with a period of 0.1 s; guided by this prior, MPPI performs sampling and local optimization at a high frequency of 0.01 s to generate the final control commands.

**Figure 5:**
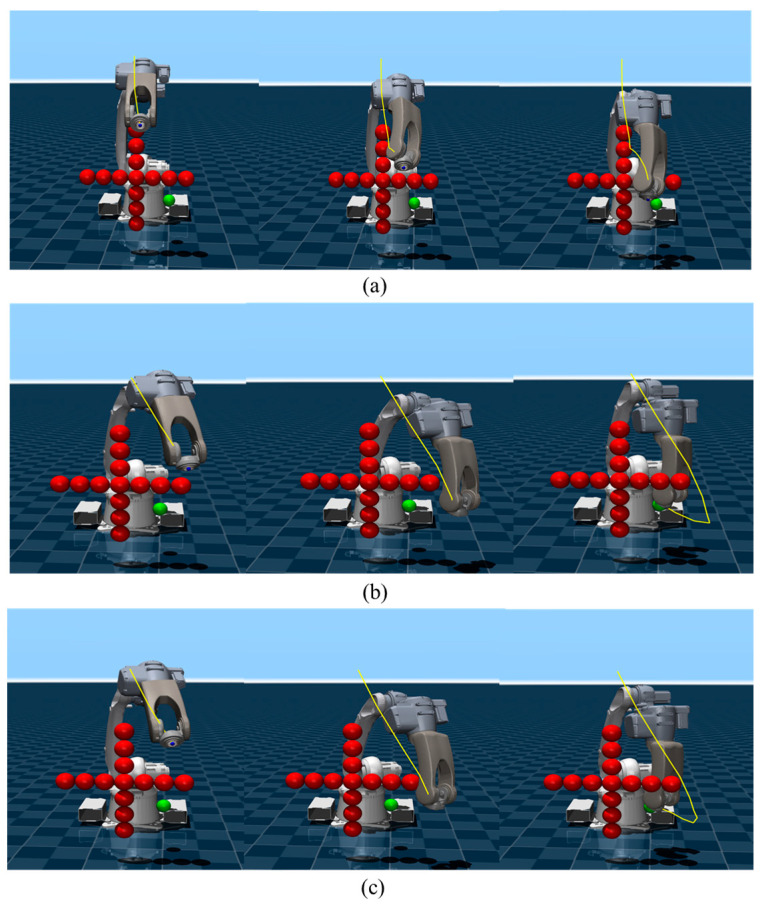
Motion trajectories in standard obstacle scenario. (**a**) SF-MPPI; (**b**) SF-SAC; (**c**) PG-MPPI.

By independently implementing the SF-MPPI, SF-SAC and PG-MPPI trajectory planning algorithms, we obtain the motion trajectories of the robot end-effector, as shown in Figure 5, where the motion trajectories are marked with yellow curves.

SF-MPPI achieves hard-constraint collision avoidance for local obstacles via the safety filter, yet its myopic optimization property makes it prone to falling into local minima in the absence of long-horizon guidance. This is manifested by the trajectory terminating non-targeted in front of obstacles, thus failing to complete the end-effector positioning task. In contrast, both SF-SAC and PG-MPPI have internalized the prior of obstacle distribution in the training environment and are able to plan globally optimal obstacle avoidance paths. The experimental results show that these two algorithms can not only reach the target position accurately but also maintain a safe distance between all links of the manipulator and obstacles throughout the entire motion process.

**Figure 6:**
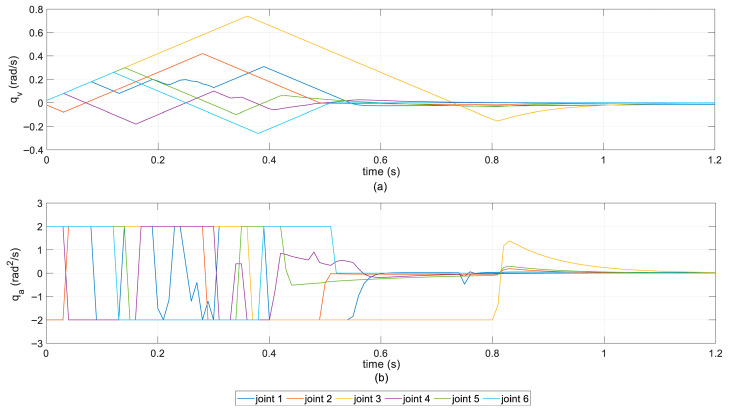
Joint motion curves of PG-MPPI. (**a**) Joint velocity; (**b**) joint acceleration.

The joint kinematic response curves of the PG-MPPI algorithm are shown in Figure 6. Throughout the entire motion cycle, the velocity and acceleration of all joints are strictly constrained within the preset physical boundaries, with no constraint violations. Meanwhile, the velocity trajectories exhibit a continuous and smooth characteristic, with low fluctuations, verifying that the proposed algorithm ensures physical feasibility while achieving excellent motion smoothness.

**Figure 7:**
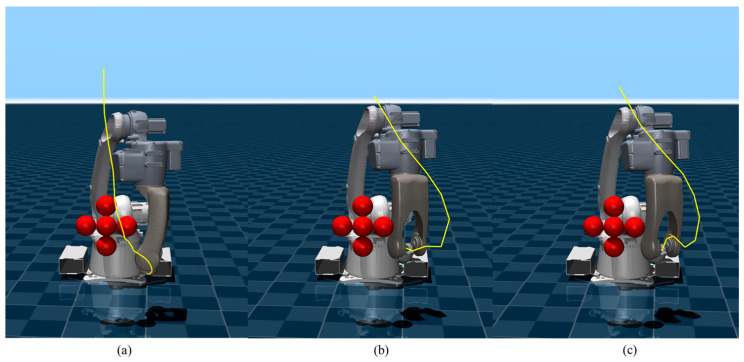
Motion trajectories in simplified obstacle scenario. (**a**) SF-MPPI; (**b**) SF-SAC; (**c**) PG-MPPI.

A simplified obstacle scenario is presented in Figure 7, where all three algorithms achieve collision-free target arrival. Due to the lack of global policy guidance, SF-MPPI can only complete path planning through local obstacle avoidance, resulting in a narrow safety margin between the trajectory and obstacles. In contrast, SF-SAC and PG-MPPI leverage the offline-trained global planning prior to generate obstacle avoidance trajectories with sufficient safety margins and reach the target position successfully.

**Figure 8:**
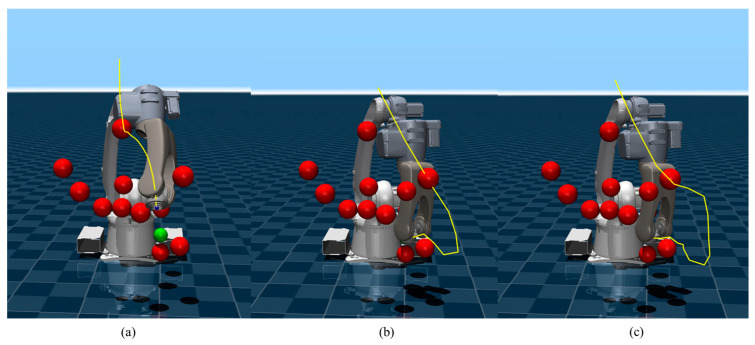
Motion trajectories in complex obstacle scenario. (**a**) SF-MPPI; (**b**) SF-SAC; (**c**) PG-MPPI.

A reconfigured obstacle layout based on the standard environment is shown in Figure 8, where both SF-MPPI and SF-SAC expose their inherent limitations: limited by local optimization bias, SF-MPPI tends to get trapped in dead zones in obstacle-dense areas, leading to task failure; the offline policy of SF-SAC suffers from generalization failure due to the unseen obstacle distribution, resulting in trajectory collisions. On the contrary, through the collaborative mechanism of global policy guidance and local online optimization, PG-MPPI not only avoids potential collision traps using prior knowledge but also makes adaptive adjustments to un-modeled obstacles via real-time iteration of MPPI, thus generating a collision-free feasible trajectory and successfully completing the positioning task.

To quantitatively evaluate the generalization and obstacle avoidance performance of the three algorithms, we randomly generate 10 typical initial joint configurations of the manipulator and test them in both the standard and complex obstacle simulation environments. We evaluate the collision-free target arrival success rates and planning time of the three algorithms, with the results presented in Table 3.

In the standard obstacle environment used for training, both SF-SAC and PG-MPPI achieve a 100% collision-free success rate with comparable performance. However, in the more challenging complex obstacle environment, PG-MPPI still maintains a 100% success rate by virtue of the collaborative mechanism of global policy guidance and local real-time optimization, outperforming the SF-SAC algorithm significantly. The above results fully demonstrate that the proposed algorithm has superior environmental generalization ability and dynamic obstacle avoidance performance.

In terms of planning efficiency, SF-SAC exhibits the lowest planning time (1.14 ± 0.12 ms) owing to its forward inference. SF-MPPI requires 1.47 ± 0.4 ms for local sampling and optimization. PG-MPPI consumes slightly more time (3.03 ± 0.82 ms) because it integrates offline policy guidance, MPPI sampling optimization, and CBF safety filtering. Nevertheless, all three methods run within 5 ms, fully satisfying the real-time requirements for online trajectory planning of industrial manipulators.

**Figure 9:**
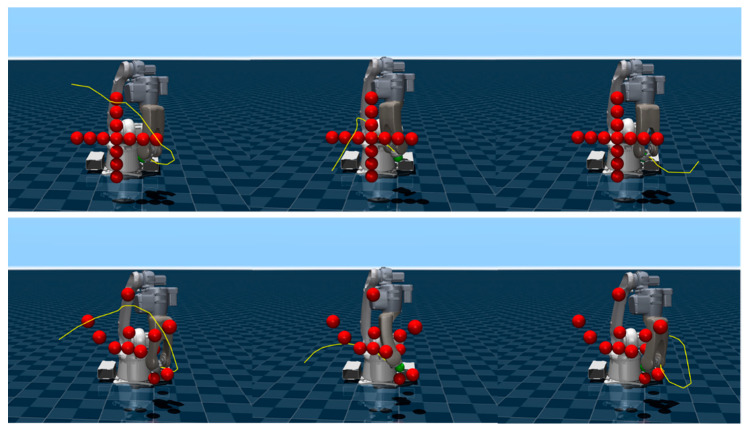
Motion trajectories based on PG-MPPI.

Some representative trajectories planned by the PG-MPPI algorithm in the above experiments are presented in Figure 9. Multiple trajectories generated for different initial joint configurations can flexibly adapt to the obstacle distribution in the environment, generate collision-free paths with sufficient safety margins, and guide the manipulator to reach the target position accurately. This fully demonstrates the algorithm’s robustness in trajectory planning and environmental adaptability under different initial conditions.

Based on the above qualitative and quantitative experimental analyses, the PG-MPPI algorithm proposed in this paper achieves global obstacle avoidance while completing local obstacle avoidance. Moreover, through the dual guarantee of the constraint discounting mechanism and safety filtering, it imposes hard constraints on the velocity and acceleration of joint trajectories, effectively ensuring the smoothness and stability of the manipulator’s motion trajectory. This algorithm demonstrates strong practical potential for industrial deployment. Its hierarchical architecture shifts most of the computational burden to the offline stage, leaving only lightweight sampling-based optimization and CBF filtering to be executed online, which is well aligned with the real-time control requirements of industrial manipulators. At the same time, it supports sim-to-real adaptation strategies, such as dynamics randomization and network light weighting. Moreover, its multi-layer safety framework can more rigorously prevent production risks such as collisions and joint-limit violations.

## 5. Conclusions

Aiming at the problems of manipulator motion planning in complex environments with unknown obstacle configurations, this study proposes a PG-MPPI algorithm that integrates global policy guidance and local model predictive optimization. By incorporating a pre-trained CD-SAC policy network, the PG-MPPI algorithm provides a high-quality initial sampling distribution for the MPPI module, which effectively addresses the problems of low sampling efficiency and susceptibility to local optima for traditional MPPI in high-dimensional spaces. Simulation results show that, across multiple static and reconfigurable obstacle scenarios designed in this paper, the proposed PG-MPPI algorithm achieves superior or comparable performance to the SF-SAC and SF-MPPI baselines in terms of collision-free success rate, planning efficiency, and trajectory smoothness. This validates the effectiveness and generalization capability of the proposed method for high-dimensional manipulator motion planning tasks. Future work will further explore the deployment of this framework on physical hardware platforms and its integration with an environmental perception module to enable real-time adaptive planning for unknown dynamic obstacles.

## Figures and Tables

**Table 1:** CD-SAC Parameters.

Type	Parameters	Value	Function
Constraint Discount	velocity bound	1 rad/s	Joint velocity constraint
	acceleration bound	2 rad^2^/s	Joint acceleration constraint
	$p^{\max}$	1	Termination probability calculation in Equation (19)
	$\tau_{c}$	0.99	Exponential moving average decay rate in Equation (20)
SAC Network	hide dim	256	Number of neurons per layer for actor/critic MLP
	learning rate	3 × 10^−4^	Learning rate of Adam optimizer
	γ	0.99	Baseline discount factor of SAC in Equation (21)
	batch size	256	Batch size for each network update
	action repeat	5	Number of physics simulation steps per RL step
Environment Reward	reach tolerance	0.03	Position error threshold for task success
	max ep steps	2500	Maximum steps per training episode
	success bonus	10.0	Extra reward
	total steps	200,000	Total environment interaction steps

**Table 2:** PG-MPPI parameters.

Parameters	Value	Function
policy update	0.1 s	Update cycle of the global prior policy
horizon	25	Length of the predictive receding horizon
samples number	200	Number of sampled trajectories for MPPI
λ	0.6	Temperature parameter
standard noise	0.6	Standard deviation of Gaussian noise

**Table 3:** Results of typical initial configurations.

Method	Normal SR (%)	Complex SR (%)	Planning Time (ms)
SF-MPPI	20	20	1.47 ± 0.4
SF-SAC	100	60	1.14 ± 0.12
PG-MPPI	100	100	3.03 ± 0.82

## Data Availability

The experimental data and source code associated with this study are available at the following repository: https://github.com/FantasyRobot/rl_mppi (accessed on 23 March 2026).
